# Chronic cholestasis detection by a novel tool: automated analysis of cytokeratin 7-stained liver specimens

**DOI:** 10.1186/s13000-021-01102-6

**Published:** 2021-05-06

**Authors:** Nelli Sjöblom, Sonja Boyd, Anniina Manninen, Anna Knuuttila, Sami Blom, Martti Färkkilä, Johanna Arola

**Affiliations:** 1 Department of Pathology, University of Helsinki and Helsinki University Hospital, Haartmaninkatu 3, 00290 Helsinki, Finland; 2 Aiforia Technologies Oy, Tukholmankatu 8, 000290 Helsinki, Finland; 3 Department of Gastroenterology, University of Helsinki and Helsinki University Hospital, 00290 Helsinki, Finland

**Keywords:** Artificial intelligence, AI model, Machine learning, Primary sclerosing cholangitis, Cholestasis, Liver histology

## Abstract

**Background:**

The objective was to build a novel method for automated image analysis to locate and quantify the number of cytokeratin 7 (K7)-positive hepatocytes reflecting cholestasis by applying deep learning neural networks (AI model) in a cohort of 210 liver specimens. We aimed to study the correlation between the AI model’s results and disease progression. The cohort of liver biopsies which served as a model of chronic cholestatic liver disease comprised of patients diagnosed with primary sclerosing cholangitis (PSC).

**Methods:**

In a cohort of patients with PSC identified from the PSC registry of the University Hospital of Helsinki, their K7-stained liver biopsy specimens were scored by a pathologist (human K7 score) and then digitally analyzed for K7-positive hepatocytes (K7%area). The digital analysis was by a K7-AI model created in an Aiforia Technologies cloud platform. For validation, values were human K7 score, stage of disease (Metavir and Nakunuma fibrosis score), and plasma liver enzymes indicating clinical cholestasis, all subjected to correlation analysis.

**Results:**

The K7-AI model results (K7%area) correlated with the human K7 score (0.896; *p* < 2.2e^− 16^). In addition, K7%area correlated with stage of PSC (Metavir 0.446; *p* < 1.849e^− 10^ and Nakanuma 0.424; *p* < 4.23e^− 10^) and with plasma alkaline phosphatase (P-ALP) levels (0.369, *p* < 5.749e^− 5^).

**Conclusions:**

The accuracy of the AI-based analysis was comparable to that of the human K7 score. Automated quantitative image analysis correlated with stage of PSC and with P-ALP. Based on the results of the K7-AI model, we recommend K7 staining in the assessment of cholestasis by means of automated methods that provide fast (9.75 s/specimen) quantitative analysis.

**Supplementary Information:**

The online version contains supplementary material available at 10.1186/s13000-021-01102-6.

## Background

A core needle biopsy specimen from the liver is the gold standard for the diagnosis of liver diseases and is considered particularly important in the assessment of inflammatory activity and stage of fibrosis [1]. Cytokeratin 7(K7) is a common immunohistochemical (IHC) marker for chronic cholestasis in liver biopsies - especially in liver diseases with biliary tract inflammation [2]. In the normal liver, K7 expression occurs in the biliary epithelium, whereas hepatocytes remain negative. However, in chronic cholestasis, periportal hepatocytes and intermediate hepatobiliary cells (progenitor cells) stain positive for K7 [3, 4]. Thus, bile duct loss and chronic cholestasis can be distinctly elucidated via K7 stain [5]. Moreover, the K7 stain correlates with the biochemical markers of cholestasis: plasma alkaline phosphatase (P-ALP) and plasma bilirubin (P-Bil), and a biomarker of cytolytic activity: plasma alanine aminotransferase (P-ALT) [6, 7]. Some studies indicate that the K7 stain should be considered as a prognostic marker for rapidly progressive chronic cholestatic cholangiopathies [6–9]. One caveat with the K7 analysis—as with any image analysis based on an evaluation conducted by an individual pathologist—is, however, that it is not entirely objective, having intra- and interobserver variability [10]. In addition, it would be presumably very time-consuming for a human being to calculate in a histological sample all the cells of a certain type.

Automated image analysis is a potential solution for this problem. Artificial Intelligence (AI) by convolutional neural networks (CNNs) is a powerful technology for pattern recognition and image classification [11, 12]. Additionally, it is a suitable instrument for medical image analysis in various fields, including pathology. It displays the potential to improve performance of the analysis in certain applications [13, 14]. CNNs are machine-learning algorithms that require image data and labels as input, from which the CNN learns and extracts image features. In the case of supervised learning, the CNN is trained by using human-made training annotations [11, 15]. In histopathology, this means that a human being annotates desired features by labelling digital images of tissues [13, 14]. In the case of semantic segmentation, the annotations provided by a human being are pixel-level image segments that represent the classes to be learned by the CNN (for example hepatocytes or portal tracts). The output data of a CNN consists of interpretation and classification of images based on the features learned from the input data [11, 12, 15].

We aimed to build a CNN-based AI model for the automated detection of K7-positive hepatocytes (K7-AI model). To our knowledge, such digital tools have not been previously studied in assessment of chronic cholestasis. Nested layer structures of CNNs have proven their excellence in fully automated complex image analysis tasks compared to the traditional computer vision [12, 16, 17]. Thereby, we aimed to explore the potential of CNNs, and the commercial cloud-based Aiforia® platform in this particular setting. To validate the AI model and its results, we utilized a traditional scoring method performed by a liver pathologist (human K7 score) of K7-stained whole-slide images (WSIs). To further test the performance of the K7-AI model within our cohort of patients with PSC, we studied the K7 load in liver tissue and its correlation with first, the amount of fibrosis, and second, the P-ALP levels.

## Methods

### Patient cohort and liver biopsies

The department of gastroenterology at Helsinki University Hospital (HUH) maintains a 2010-founded registry of a PSC patient cohort, a registry including clinical and laboratory parameters, ERC (endoscopic retrograde cholangiography) scores, cytology, and histology reports from almost 1000 PSC patients. The PSC diagnosis of the cohort utilized here was primarily based on magnetic resonance cholangiopancreatography (MRI-MRCP) or on ERC. A histological liver specimen (from a core-needle biopsy) is retrieved from most of the PSC patients in our center to exclude concomitant autoimmune hepatitis and to confirm the diagnosis in cases with mild intrahepatic disease in imaging (by ERC). The cohort of this study consists of patients with liver biopsies performed between 1988 and 2018.

The rationale behind our choice of this particular patient cohort is the specific histological appearance of PSC in the liver tissue as the disease progresses. PSC is a chronic progressive cholangiopathy [18] resulting in cholestasis, and cholestasis, when chronic, is indicated by K7-positive hepatocytes in a liver specimen [6–9].

For clinical data on our patient cohort, see Table 1. In 318 patients, we did 366 liver biopsies (1988–2018), and 207 of them provided representative K7-stained liver specimens numbering 210.

**Table 1:** Primary sclerosing cholangitis (PSC) patient cohort and liver biopsies

Number of patients	318
Number of patients, male	182 (63%)
Age at diagnosis in years, mean, (range)	34.9 (10–75)
Duration of disease (years) at liver biopsy, mean (range)	3.26 (0–25)
Stage of fibrosis (Metavir, 0–4) in liver specimen, mean, (range)	1 (0–4)
Biopsies with stage 0 (Metavir) fibrosis, %	44.75
Biopsies with stage 1 (Metavir) fibrosis, %	24.59
Biopsies with stage 2 (Metavir) fibrosis, %	19.14
Biopsies with stage 3 (Metavir) fibrosis, %	8.33
Biopsies with stage 4 (Metavir) fibrosis, %	3.19
Plasma ALP (U/l) levels ±3 months from liver biopsy, mean ± SD, (range)	216.75 ± 198.38 (42–1514)

Written informed consent was obtained from each patient included in the study. The study protocol confirms to the ethical guidelines of the 1975 Declaration of Helsinki as reflected in a priori approval by the institution’s human research committee (license number 278/13/03/01/09, Ethical Statement of the Internal Medicine § 305, HUH).

### Liver biopsies and immunohistochemistry

All liver biopsies were acquired under ultrasound guidance as part of a routine diagnostic liver-disease workup. All specimens were fixed with formalin. Two of the most important staining methods applied for routine liver-biopsy diagnostics in our laboratory (Diagnostic Centre, HUH, Dept. of Pathology) include the histochemical Herovici stain to highlight collagen and fibrosis in liver tissue, and the K7 immunohistochemical stain to highlight bile-duct epithelium and cholestatic hepatocytes.

We performed K7 staining of the biopsy specimens in the pathology laboratory of the Diagnostic Centre, HUH, according to the standard staining protocol. Three-micrometer sections were cut from the formalin-fixed paraffin-embedded (FFPE) needle biopsy specimens. A Ventana Benchmark Ultra instrument (SP52, Roche 790–4462; Roche, Tucson, AZ, USA) served as the staining instrument for the K7 antibody. Pre-treatment was done with Cell Conditioning 1 buffer, pH 8.5, (Roche 950–124), for 64 min at 98 °C. Incubation for K7 antibody was 16 min at 37 °C. A multimer-based detection kit, Ultraview (Roche 760–500), served for detection of the K7 antibody (8 min at 37 °C). The reaction was visualized with DAB (3,3′-diaminobenzidine, Roche 760–500), and sections were counterstained with hematoxylin.

### Imaging and software

We acquired the digital WSIs of the K7-stained and Herovici-stained histological slides by means of a Pannoramic SCAN II 150 (3DHistech) scanner. The object magnification was 20x, and the original pixel size was 0.221 μm. The WSIs were uploaded to Aiforia® Cloud, a commercial cloud-based platform for managing, sharing, and viewing WSIs as well as for training CNNs for automated image analysis of WSIs. If the histological slide included more than a single needle biopsy section, we manually assigned a region of interest (ROI) to avoid double-analysis. The ROI selected for the analysis contained an entire section of the needle biopsy specimen to avoid selection bias.

### CNN training – creating the K7-AI model via supervised learning

**Fig. 1:**
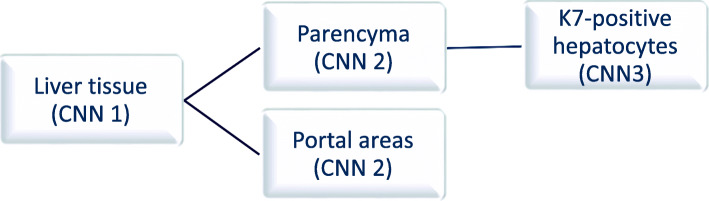
The structure of the final AI model. The three independent and nested CNNs that were trained by semantic segmentation are 1) liver tissue, 2) parenchyma and portal areas and finally 3) the K7-positive hepatocytes. Ductular proliferation and original bile ducts are trained and included in the portal areas’ layer

We trained the same CNN algorithm using three independent datasets for supervised semantic segmentation of the liver histology and quantitation of K7-positive hepatocytes. The final K7-AI model included all three independent and nested CNNs, all trained in the Aiforia platform. For the final structure of the AI model see Fig. 1. The first CNN model was trained to classify liver tissue. The second model was trained to segment liver parenchyma and portal areas within the liver tissue segment. The third model was trained to classify K7-positive hepatocytes within the parenchyma segment. The periportal ductular reaction and possible small bile ducts within the parenchyma have been trained to the AI model and segmented in the portal areas. Thus, the AI model will be able to separate K7-positive hepatocytes from the bile duct epithelium, since they are located in different classes within the CNN.

During model inference, the three CNNs were run as a single pipeline on the WSIs. The full training dataset, including 70 WSIs selected from the whole cohort of 210 WSIs, was intended to represent all variation in the cohort. The ground truth for annotating the different image features in the training material (70 WSIs) is in Additional file 1: Table 1. The input data which constitutes the training annotations made in the training set (training regions) are in Additional file 1: Table 2. An independent set of 57 cases from 210 WSIs was used for validation purposes and excluded from the training material. The remaining 83 WSIs were later analyzed by the complete K7-AI model and included in the correlation analysis demonstrated in the results.

**Fig. 2:**
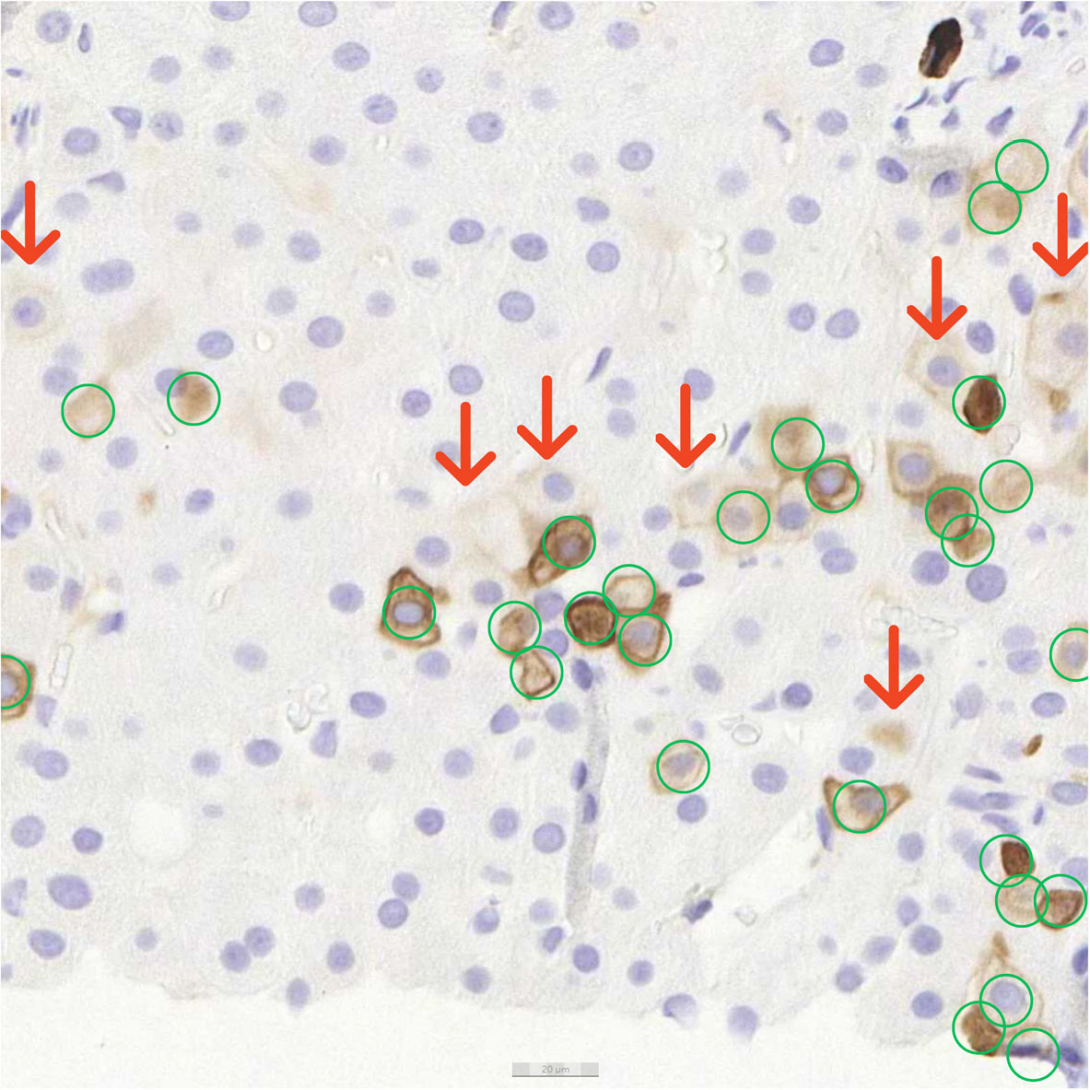
Cut-off staining intensity for K7-positive hepatocytes. Cells pointed with red arrows are considered negative, because they are stained too lightly. Thus, they are excluded from the training annotations made for the AI model. The darker brown hepatocytes circled in green were considered positive. They were annotated in the training phase to demonstrate and teach the AI model what K7-positive hepatocytes look like

The cutoff for positivity of hepatocytes in the K7 stain is in Fig. 2. The cells pointed with red arrows are stained only lightly and were excluded from the annotations in order to emphasize K7-positive hepatocytes for the AI model. The hepatocytes stained deeper brown (in Fig. 2) and circled with green were considered positive and taught to the AI model as positively stained hepatocytes. Staining intensity varies in the image material to some extent, due to the age differences among biopsy specimens. The real-world variance of the feature of interest should be presented as well as possible in the training data in order to achieve a high-performing CNN. Thus, including older biopsy specimens with potential fading in the IHC stain in the training set led to development of a model able to manage the analysis of older sections.

The perceptive field diameter (field of view) applied was 100 μm for the liver tissue layer, 50 μm for the portal areas and liver parenchyma layer, and 20 μm for the K7-positive hepatocyte layer. The training data was enhanced by the image augmentation presented in Additional file 1: Table 3. All training data and a maximum of 20,000 iterations served in the final training for all CNNs. If training loss was not reduced in 200 consecutive iterations, training automatically came to a halt.

### Validation of K7-AI model performance

To independently validate the performance of the final and complete K7-AI model, meaning its ability to detect features it had not previously encountered in the training set, a board-certified pathologist and specialist in liver diseases (S. Boyd) performed human K7 scoring. The scoring was performed for 57 WSIs that were separated from the training material as an independent validation set. This validation set constituted altogether 27.1% of the whole image material (210 images). The human K7 score was performed according to the protocol in Table 2 [19]. Well-established scoring systems are lacking for PSC. Thus, we applied the aforementioned protocol in our study, originally designed for scoring liver specimens of patients with primary biliary cholangitis (PBC), for its prognostic properties validated specifically in a PSC liver specimen cohort in an international multi-center study [20]. The K7-AI model’s results (K7%area) in the validation set of 57 WSIs were then correlated with the human K7 score. In addition to the validation set, the human K7 score was evaluated for the whole material (210 specimens) by applying the protocol in Table 2 [19]. Finally, the human K7 score and the K7-AI model’s results (K7%area) were correlated to confirm K7-AI model performance.

**Table 2:** Deposition of K7 in hepatocytes in a liver biopsy specimen and stage of fibrosis according to Nakanuma classification. Deposition of orcein-positive granules in Nakanuma classification has been replaced with a similar model, along with applying the deposition of K7-positive hepatocytes in the evaluation of chronic cholestasis. Adapted from [Nakanuma, Y., Zen, Y., Harada, K., Sasaki, M., Nonomura, A., Uehara, T., et al. [19]. Application of a new histological staging and grading system for primary biliary cirrhosis to liver biopsy specimens: Interobserver agreement. *Pathology International, 60*(3), 167–174, [19]

**Deposition of K7-positive hepatocytes (score 0–3)**	**Scoring protocol**
0	No K7-positive hepatocytes
1	K7 positivity in at least ten hepatocytes in one periportal area (zone 1)
2	K7 positivity in at least ten hepatocytes in 1/3–2/3 of periportal areas
3	K7 positivity in at least ten hepatocytes in more than 2/3 of periportal areas
**Nakanuma classification - stage of fibrosis in a liver biopsy specimen**	
**Stage of fibrosis (0–3)**	**Scoring protocol**
0	No portal fibrosis or fibrosis limited to portal tracts
1	Portal fibrosis with periportal fibrosis or incomplete septal fibrosis
2	Bridging fibrosis with variable lobular disarray
3	Liver cirrhosis with regenerative nodules and extensive fibrosis

### Comparison of the K7-AI model’s results (K7%area) with biochemical examinations and with disease stage

The entire material (210 WSIs) was analyzed with the K7-AI model. Image data analyzed by the K7-AI model was examined categorically from the specimens in the following order: 1. area of liver tissue, 2. area of parenchyma and portal areas and their proportions of the liver tissue, and 3. proportion (%) of K7-positive (cholestatic) hepatocytes from the parenchyma (K7%area).

Validation of the K7-AI model’s results required comparing analysis of the AI model with the human K7 score. As a complementary validation analysis, we also compared the K7-AI model’s results with fibrosis (including both Metavir- and Nakanuma-stage) scores, as well as with the clinical data of the cohort extracted from the PSC registry.

According to the literature, in a PSC liver biopsy specimen, the amount of fibrosis highlights the progression of the disease [20–23]. Thus, for validation purposes, a board-certified liver pathologist (S. B) performed histological scoring of fibrosis scores according to the Nakanuma classification [19, 24] from the Herovici-stained WSIs of the same cohort (Additional file 2: Fig. 1). See in Table 2 the formula for the Nakanuma fibrosis score [19]. In addition, original structured histological reports, ones serving previously for clinical purposes, were included in fibrosis evaluation. All liver biopsies in our laboratory receive estimated Metavir scores from 0 to 4 depending on amount of tissue fibrosis [25]. Metavir scores from the PSC registry for the biopsy specimens in this study had all been evaluated by a number of liver pathologists in our laboratory between 1988 and 2018. Metavir score was available for 182 biopsy specimens.

The objective was also to measure whether one scoring method (Nakanuma vs Metavir) would outperform the other when calculating correlations between fibrosis stage and analysis of the K7-AI model (K7%area).

Clinical data included P-ALP level measurements (U/l) (*n* = 112). These levels correlate well with clinical stage of cholestasis [21]; here, the levels were measured as close as possible of the biopsy date (± 3 months). The other clinical markers of chronic cholestasis, such as plasma gamma-glutamyl transferase (P-GT) U/l, along with P-ALP levels, were compared to our AI model’s analysis (K7%area) to learn whether these parameters showed any correlation.

### Statistical analysis

Analysis data (K7%area of each biopsy) of the 210 images was exported from Aiforia in one format of a CSV file. All the statistics were computed using R (Version 3.5.2; R Foundation for Statistical Computing, Vienna, Austria, 2018) and the ggplot2 (Hadley Wickham, Springer-Verlag, New York, NY, USA 2016) package.

Spearman coefficient correlation were calculated to examine the correlation between laboratory parameters and K7%area, and between fibrosis stage and K7%area. Pearson correlation coefficient were calculated to examine the correlation between laboratory parameters and K7%area, and between stage of fibrosis and K7%area. A natural logarithmic (ln) transformation performed on the continuous variables met the normality assumption of the Pearson correlation coefficient test. We considered correlations significant at *p* ≤ 0.05.

## Results

### K7-AI model ready to go

**Fig. 3:**
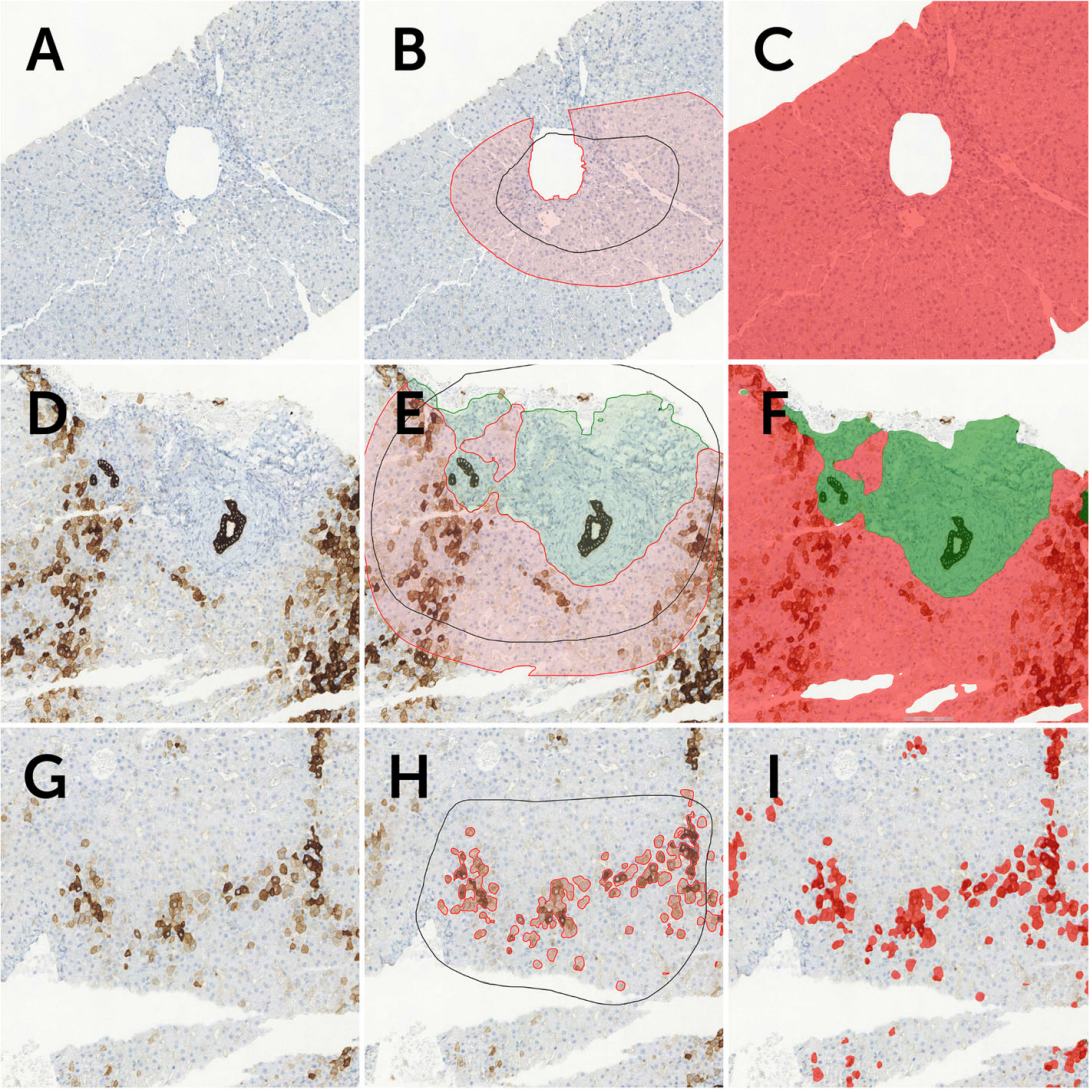
**a** A standard cytokeratin 7 (K7) immunohistochemical stain of a liver core biopsy. **b** The black circle illustrates the training region, the red circle the annotation region, both drawn by a pathologist to teach the AI model to recognize liver tissue in the image. Space within the black circle but outside the red circle is considered background and not tissue. **c** The red area is what the K7-AI model considers liver tissue. **d** The same K7 stain of the same liver biopsy specimen with **e** training annotations drawn by a pathologist to teach the AI model the difference between portal areas and liver parenchyma. Within the black training regions, circled green areas represent the portal areas and red-circled areas represent parenchyma. **f** K7-AI model inference mask, meaning the interpretation of the AI model of the specimen. Accordingly, green areas are what the AI model considers as portal areas and red areas are liver parenchyma. **g** Notice the multiple cholestatic DAB-positive hepatocytes in this K7-stained biopsy specimen. **h** Red annotation regions within the training regions (black) are the areas taught to the AI model as K7-positive hepatocytes within the liver parenchyma. **i** The red areas are what the K7-AI model interprets as K7-positive hepatocytes after training

Our results, reflecting how well the K7-AI model had learned the features in opposition to the training annotations, were examined after each training round. For the K7-AI model’s visual interpretation (visual results) of histological images see Fig. 3. Results were based on the features annotated in the images in the training phase. The total areas of errors in each layer of the AI module are in Table 3, along with the precision and sensitivity percentages for segmentations in each layer.

**Table 3:** Total area errors, precisions and sensitivities per each layer. Since there are two classes in the portal areas and parenchymal layer, highest class-specific errors have been evaluated for both classes. Total area errors are the sum of false positive (FP) and false negative (FN), namely the total errors per training areas in each layer of the AI model

	Layer for Liver Tissue	Layer for Portal areas & Parenchyma^a^	Layer for K7 positive hepatocytes
Total area error (%)	0.56	2.6	0.24
Precision of the segmentation (%)	99.4	98.4	92.9
Sensitivity of the segmentation (%)	99.6	98.3	91.8
Highest class-specific error accepted per class % (FP%/FN%^1^)	1.02% (0.63%/0.39%)	2.26% (1.03%/1,23%)^2^, 6.82% (3.42%/3.40%)^3^	15.24% (7.00%/8.24%)

^a^Since there are two classes in the portal areas and parenchymal layer, highest class-specific errors were evaluated for both classes

Errors in CNN inference may raise the false-negative error of the subsequent CNN, because the previous CNN clips the region of the subsequent CNN; training annotations for the subsequent CNN outside the clipping mask of the previous CNN are therefore undetectable. The highest class-specific errors are illustrated in Table 3.

### Analysis of the validation set – 57 images

Performance of the K7-AI model was independently validated in a set of 57 WSIs before our analysis of the entire cohort (210 WSIs). The total time to complete inference of the full K7-AI model pipeline with three CNNs for the 57 WSIs was 9 min, 16 s, averaging 9.75 s per biopsy specimen.

In addition, we applied an external and independent validation method for the validation set by evaluating the correlation between the human K7 score and the K7-AI model’s result that showed K7%area. The protocol in Table 2 was applied in the human analysis of K7-positive hepatocytes. In Pearson’s product moment correlation, the ln (K7%area) versus the human K7 score was 0.92 (*p*-value < 2.2e-16). In Spearman’s rank correlation, the result was 0.929 (p-value < 2.2e-16).

### Correlation of K7-AI model results with human K7 score in the whole PSC cohort (210 WSIs)

**Fig. 4:**
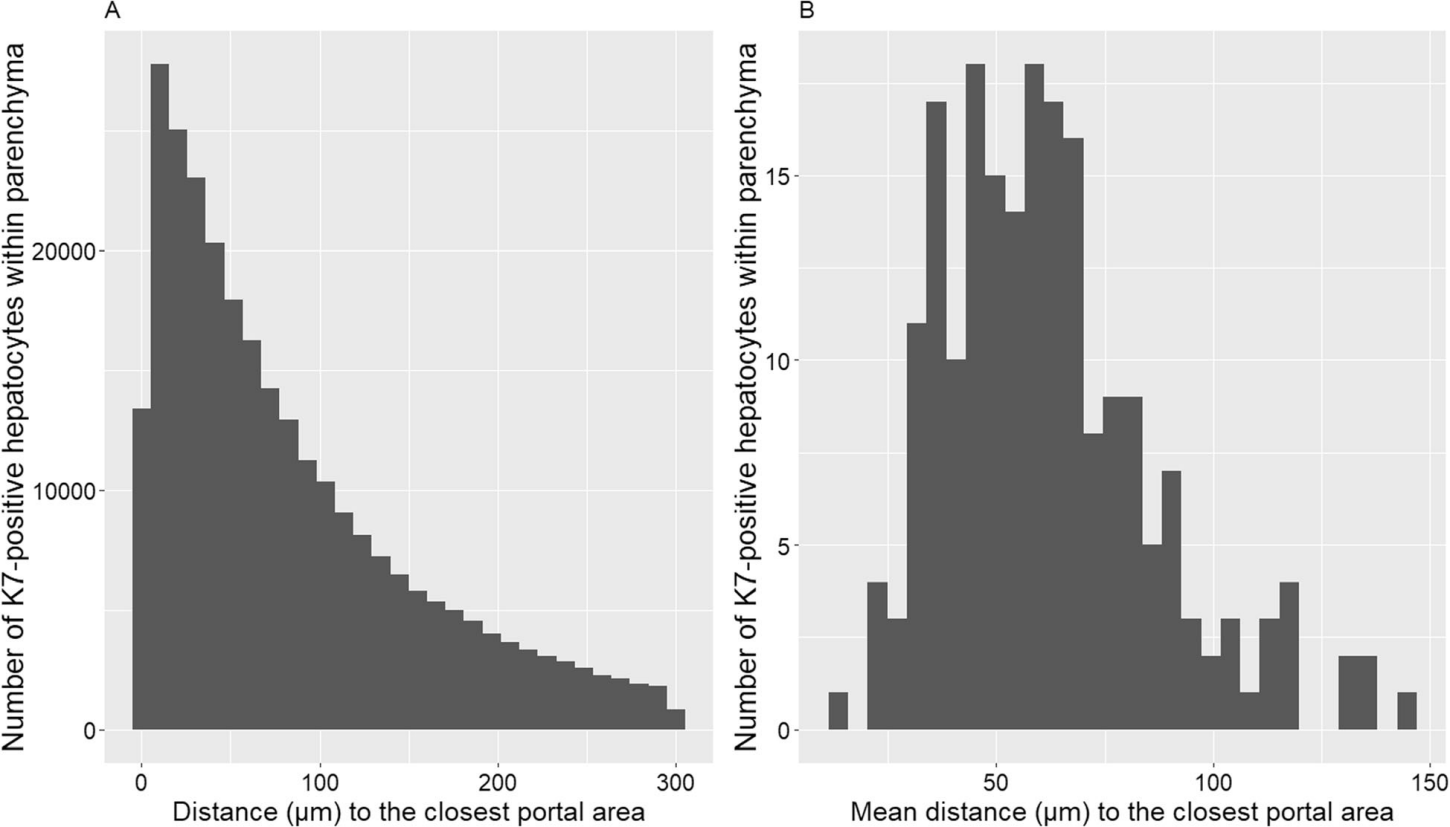
**a** The distribution of K7-positive hepatocytes in length (μm) from the closest portal area **b** The mean distance (μm) of K7-positive hepatocytes of the closest portal area (both in the entire material of 210 slides)

To be able to compare the human K7 score with K7%area and evaluate their correlation, we also applied the K7 AI Model to calculate the number of K7-positive hepatocytes and their distribution in each specimen. The normal length of portal sinusoids in human liver is 447–721 μm [26]. Zone 1, which is the periportal area, is the third of the length of the portal sinusoids [26]. Figure 4 illustrates the absolute distance (A) (μm) and mean distance (B) (μm) of K7-positive hepatocytes from the closest portal area. The majority of the K7-positive hepatocytes are located in zone 1. Spearman’s rank correlation between the K7%area and the mean distance (μm) of K7-positive hepatocytes from the nearest portal area was 0.44 (*p*-value < 6.4e-11).

**Fig. 5:**
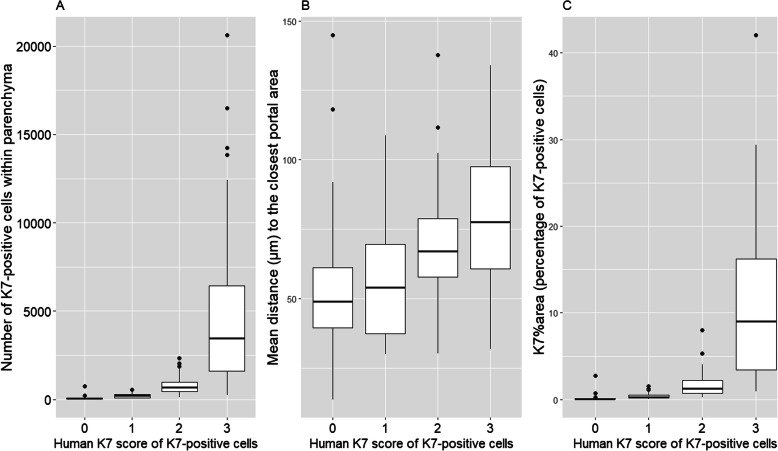
**a**-**c** The human estimate of the K7-positive hepatocytes (human K7 score) binned into three categories (0–3) according to the Nakanuma classification [19] in relation to the distribution of K7-positive hepatocytes measured by the K7-AI Model. **a** Number of K7-positive hepatocytes and their distribution within the categories of human K7 score (0–3) estimated by a human pathologist. **b** Mean distance (μm) of the K7-positive hepatocytes from their closest portal area and their distribution within the categories of human K7 score. **c** Percentage of area of K7-positive hepatocytes (K7%area) in each specimen distributed within the categories of human K7 score

Because the human K7 score is a categorical variable, we also calculated the distribution of the K7-positive hepatocytes in relation to human K7 score (0–3) (Fig. 5). The more cholestasis and K7-positive hepatocytes, the further the K7-positive hepatocytes are located from the closest portal area.

**Fig. 6:**
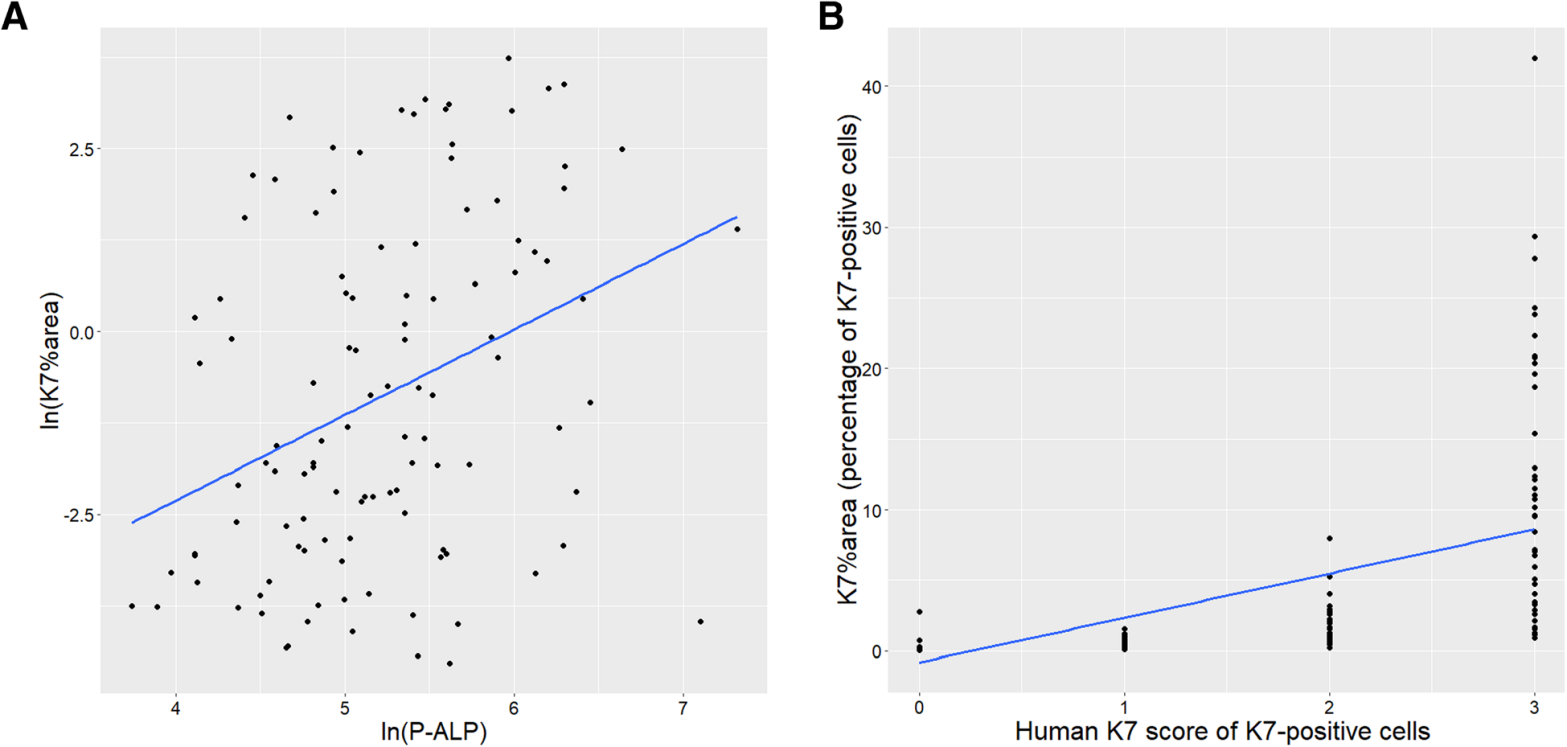
The K7-AI model’s results (K7%area) vs the human estimate of K7-positive hepatocytes (human K7 score), and the logarithm of the K7-AI model’s results versus serum ln (ALP) values

The K7-AI model’s percentage of K7-positive hepatocytes in the liver parenchyma (K7%area) correlated well with the human K7 score (Fig. 6). Pearson’s product moment correlation between K7%area and the human K7 score was 0.615 (*p* < 2.2e-16). The correlation between the ln (K7%area) and the human K7 score was 0.907 (p < 2.2e-16) which indicates a very strong correlation. Spearman’s correlation was 0.897 (p < 2.2e-16), also very strong.

### Correlation of K7 load in liver tissue with biochemical parameters

For overall results see summary in Table 4. For correlations of P-ALP (U/l) (*n* = 112) and K7-AI-model K7%area see Fig. 6. The Pearson correlation of log-transformed variables was weak but statistically highly significant (0.348, *p* < 0.000171). The Spearman correlation of the P-ALP levels with the K7%area was 0.359 (*p* < 1.001e-5).

**Table 4:** The K7-AI model’s results (K7%area) and their correlations with biochemical examinations indicating cholestasis, and the K7-AI model results (K7%area and ln (K7%area)) in correlation with stage of fibrosis, according to Metavir and Nakanuma classifications

**Laboratory parameter (plasma tests):**	**Pearson K7%area (*p*-value)**	**Spearman K7%area (*p*-value)**	**Pearson ln (K7%area) (*p*-value**)
ln (ALP) (alkaline phosphatase) (U/l)	0.290 (*p* = 0.002)	0.359 (*p* < 1.001–05)	0.348 (*p* = 1.71e-4)
ln (GT) (gamma-glutanyl transpeptidase) (U/l)	0.275 (*p* = 0.275)	0.204 (*p* = 0.112)	0.216 (*p* = 0.092)
ln (Bil) (non-conjugated bilirubin) (μmol/l)	0.264 (*p* = 0.012)	0.253 (*p* = 0.016)	0.273 (*p* = 0.009)
**Fibrosis stage:**	**Pearson-correlation K7%area;** ***p*-value**	**Spearman-correlation K7%area;** ***p*-value**	**Pearson-correlation ln (K7%area);** ***p*-value**
Nakanuma score (0–3)	0.289; *p* < 3.7e-4	0.467; *p* < 4.004e-12	0.457; *p* < 1.267e-11
Metavir score (0–4)	0.242; *p* < 9.1e-4	0.441; *p* < 3.438e-10	0.437; *p* < 5.083e-10

The P-ALP and ln(P-ALP) correlations with the human K7 score were 0.321 (*p* < 5.9e-4) and 0.394 (*p* < 1.855e-05) in Pearson’s correlation and 0.405 (*p* < 1.028e-05) in Spearman’s correlation.

P-GT, U/l failed to correlate with K7%area, but a weak correlation was detectable between non-conjugated bilirubin (Bil, μmol/l) and K7%area. The correlation between ln (Bil) and ln (K7%area) was 0.273 (*p* = 0.009) in Pearson’s product moment-correlation analysis, whereas the correlation between K7%area and ln (Bil) was 0.264 (*p* = 0.012).

Weak correlations were observable between In (K7%area) and plasma transaminase levels, In (P-ALT) and ln (P-AST) (each 0.323; *p* < 7.2e-4).

### Stage of fibrosis correlates with K7 load in the liver tissue

The correlation between ln (K7%area) and stage of fibrosis (Metavir) was significant (Table 4) when using the Pearson correlation, 0.437 (*p* < 5.083 e-10), a correlation that was strong regardless of whether the calculation was by Metavir or Nakanuma stage. Spearman rank correlation between Metavir stage and human K7 score was 0. 367 (*p* < 3.122e-07).

Stage of fibrosis (Metavir or Nakanuma) and plasma ln (ALP) levels correlated rather weakly, although significantly. Pearson’s correlations for ln (ALP) and Metavir score and Nakanuma score were 0.204 (*p* = 0.006) and 0.216 (*p* = 0.023).

## Conclusions

Our 210 PSC patients with K7-immuno-stained histological liver samples served as a cohort exemplifying chronic cholestatic liver disease.

In partial validation of the K7-AI model, we found that K7%area correlated both with fibrosis stage demonstrating disease stage, and with the biochemical markers of chronic cholestasis, such as plasma ALP levels. Importantly, K7-AI results showed a very strong correlation with the human K7 score based on the K7 estimate of an independent liver pathologist.

Within this PSC cohort, we found a robust correlation between the K7-AI model and human K7 score. This provides evidence that the K7-AI model is not inferior to a human pathologist (at least in our study cohort), thus validating the analytical performance of the K7-AI model. As the most significant result, this focuses attention on the confidence and sensitivity that AI models offer in image analysis. Having said this, it is crucial to acknowledge that without a properly conducted validation protocol, this type of technology shows no true value in clinical practice. We therefore used several validation approaches (human score, stage, and biochemistry).

One of the major advantages of this kind of novel technology is speed of analysis. As we verified in our validation set, the time needed to run the inference of the final K7-AI model, including the three independent CNNs, was—for each K7-stained liver biopsy specimen—less than 10 s. Moreover, the automated analysis is not only fast, but it also produces detailed, consistent, and quantitative data that can easily be confirmed visually in the digital liver specimen images on the Aiforia® cloud platform. Supportive data has already emerged as to the high performance of this type of AI technology [13, 14].

The human K7 score has critical limitations; it is only an estimate of the K7 content binned into four categories (0–3). This score can vary among differing categories at different times (intra-observer variation), in addition to interobserver variation when several pathologists analyze the same specimen [10]. In contrast, the K7-AI model data is continuous, and automated WSI analysis provides quantitative and repeatable analysis of K7 histology.

As one limitation, this particular AI model to assess chronic cholestasis in a liver biopsy was built by a single pathologist; therefore, conclusions depend on a single interpretation of a histological image. When morphological alterations in the image material occur, it might lead to the pathologist’s misinterpretation of histological findings, and furthermore, alterations to the ground truth. If the ground truth is changed during the training process, the AI model will struggle to follow the rules and learn the trained features accordingly. To train an AI model with combined input of several professionals is possible, but sustaining the same accuracy and ground truth among such professionals may be too ambitious. However, the same limitation is inherent in routine clinical pathology.

The more pre-analytical variation there is in the input data, the more training the AI model requires to learn the feature space in the training data. In practice, if the histological slides do not undergo high-standard preparation or their staining intensity is unequal among the slides due to specimens’ age differences it will lead to undesirable variation in the training material. Same variation can occur if the scanning process is performed with differing equipment or optical optimization settings are overlooked [27]. Cholestasis can have influence on the cell morphology of hepatocytes producing additional variation. Due to the aforementioned variation in the image material, the AI model might require more training annotations and more examples to learn the desired features in the images.

The K7 stain in PSC liver biopsies was a marker of chronic cholestasis and a suitable surrogate end-point marker of disease progression, given its correlation with the clinical marker of cholestasis (P-ALP). Furthermore, this correlation was sustained, independent of the analyst–either a pathologist or our AI model. P-ALP correlation with human K7 score was slightly stronger compared to its correlation with K7-AI analysis, although both correlations were weak. Nevertheless, that the correlations were derived by independent methods provides clear evidence of a true dependence between P-ALP levels and K7-positive cholestatic hepatocytes.

For PSC as a chronic cholestatic liver disease, P-ALP levels and liver histology both may prove to be surrogate end-point markers for drug trials [21–23]. Correlations between cholestatic liver disease histology and its clinical markers vary to some extent, but it should be noted that these correlations have undergone study mostly in other chronic cholestatic liver diseases such as PBC [8]. In drug development, when analyzing drug trial biopsies, these kinds of objective tools like AI and analytical instruments could prove valuable. In clinical trials objective and consistent quantitative data is required especially in assessment of treatment response. Overall, the workload of pathologists in routine diagnostics is increasing world-wide, and it is essential to develop new tools to assist the pathologist and bring aid to the rapid decision making.

To the best of our knowledge, the correlation between amount of tissue K7 and stage of chronic cholestatic liver diseases like PSC has never before been subject to study. No specific histological classification system for PSC liver specimens exists thus far, although a few algorithms based on strictly clinical parameters can predict disease progression [28]. An international multi-center cohort study [20] has validated a histological scoring method for PSC liver biopsy specimens, and the Nakanuma classification showing current amount of fibrosis had the most favorable prognostic value in predicting PSC disease progression. We utilized fibrosis stage of PSC determined according to Nakanuma standards to provide clinically meaningful validation for the K7-AI model. Metavir and Nakanuma fibrosis scores showed almost equal correlations with the proportion of K7 as indicated by the K7-AI model. K7 status could therefore be considered as a possible indicator of PSC disease progression.

Debate has arisen as to whether, for assessment of continuing cholestasis, K7 immunohistochemical staining of a histological liver specimen is superior to, for example, quantifying its orcein deposits [2, 3]. Here, the K7 AI model has proven its potential in chronic cholestasis assessment in a cohort of PSC liver specimens. The benefit of applying this PSC cohort for validation of the AI model is the large number of liver specimens demonstrating nearly normal histological status and serving as control samples.

In conclusion, we can recommend K7 staining as part of routine diagnostics of histological cholestasis, because its values correlate with clinical parameters of chronic cholestasis and fibrosis stage. Our results are promising and encouraging because digital pathology and its applications, such as our AI model, are partly replacing the traditional diagnostic methods in the future [29]. However, the visual validation of a human pathologist will remain the gold standard of diagnostics. The prospect of our AI model is promising as an objective tool for rapid digital image analysis and in the future, we aim to investigate its potential as a prognostic tool within our PSC cohort. In addition, further validation with a wider international cohort would be beneficial.

## Supplementary Information

**Additional file 1.**

**Additional file 2.**

## Data Availability

The data that support the findings of this study are available from the PSC Registry, HUH, dept. of Gastroenterology but restrictions apply to the availability of these data, which were used under license for the current study, and so are not publicly available. Data are however available from the authors upon reasonable request and with permission. The image analysis data and the AI model’s results are available in cloud.aiforia.com upon reasonable request and with permission.

## Abbreviations

AI: Artificial intelligence
CNN: Convolutional neural network
ERC: Endoscopic retrograde cholangiography
FN: False negative
FP: False positive
HUH: Helsinki University Hospital
K7: Cytokeratin-7
MRI-MRCP: Magnetic resonance cholangiopancreatography
PBC: Primary biliary cholangitis
PSC: Primary sclerosing cholangitis
P-ALP: Plasma -alkaline phosphatase
P-ALT: Plasma alanine aminotransferase
P-Bil: Plasma bilirubin
P-GT: Plasma gamma-glutamyl transferase
ROI: Region of interest
WSI: Whole slide image
