# Supplementary material for: Chronic cholestasis detection by a novel tool: automated analysis of cytokeratin 7-stained liver specimens
Source: Diagn Pathol. 2021 May 6;16:41. doi: 10.1186/s13000-021-01102-6 (PMC8101247; doi:10.1186/s13000-021-01102-6)
Supplement: Supplementary file 1 — Additional file 1. [file 13000_2021_1102_MOESM1_ESM.docx]

**Appendix A**

**Tables 1-3. Ground truth, image augmentation parameters, and input data**

Table 1. Definition of layers (ground truth)

| **Layer** | **Definition** | **Excluded features, annotated as background** |
| --- | --- | --- |
| Liver Tissue | All that is liver tissue in the WSI | Muscle tissue, nerve tissue, and/or other soft tissue not part of the liver capsule; lumen of veins over 50 mcm in diameter |
| Parenchyma** | Hepatocytes, cytokeratine 7-positive hepatocytes, lobular inflammation | Lumen of veins over 50 mcm in diameter, liver capsule, other soft tissue |
| Portal Areas** | Portal areas that include; connective tissue, bile ducts,* portal veins and arteries, portal inflammation | Lumen of veins/arteries over 50 mcm in diameter, liver capsule |
| Cytokeratin 7-positive hepatocytes | Hepatocytes in parenchymal layer positive for cytokeratine 7 staining | Cytokeratin 7-negative hepatocytes, portal areas, liver capsule |

*Some portal areas are missing bile ducts due to progressive PSC

**Parenchymal and portal areas represent the same layer, annotations for parenchyma exclude portal areas and vice versa

Table 2. Training data/Input data, 70 histological slides

| **Layer:** | **No. of histological images in training set for annotations** | **No. of Training Regions** | **Mean no. of Training regions per Image** | **Mean no. of Training Annotations per Image** | **Total area of annotations per layer (mm²)** | **Total area of training regions per layer (mm²)** | **Total area of background per layer (mm²)** |
| --- | --- | --- | --- | --- | --- | --- | --- |
| **Liver tissue** | 44 | 228 | 5.18 | 4,48 | 28,121 | 50.89 | 22.76 |
| **Parenchyma and portal areas** | 70 | 1435 | 20.5 | 34 | 71.24 and 20.01 | 114.44 | 23.19 |
| **Cytokeratin 7-positive hepatocytes** | 68 | 655 | 9.63 | 9,56 | 16.04 | 0,31 | 15.73 |

| **Parameter** | **Minimum** | **Maximum** |
| --- | --- | --- |
| Scale | -10 | 10 |
| Luminance | -5 | 5 |
| Contrast | -5 | 5 |
| JPG compression quality | 40 | 60 |
| **Other parameters:** |  |  |
| Aspect ratio 10 |  |  |
| Maximum shear 10 |  |  |
| Maximum white balance change 1 |  |  |
| Noise 0 |  |  |
| Blur pixels, max 1 |  |  |
| Blur (%) 0.5 |  |  |

Table 3. Image augmentation parameters
