# Supplementary material for: Chronic cholestasis detection by a novel tool: automated analysis of cytokeratin 7-stained liver specimens
Source: Diagn Pathol. 2021 May 6;16:41. doi: 10.1186/s13000-021-01102-6 (PMC8101247; doi:10.1186/s13000-021-01102-6)

**Appendix B**

**Fig. 1** Fibrosis stage determined from the Herovici stained slides. The stage of fibrosis was determined from scanned slides acoording to the Nakanuma protocol *[Nakanuma, Y., Zen, Y., Harada, K., Sasaki, M., Nonomura, A., Uehara, T., et al. (2010). Application of a new histological staging and grading system for primary biliary cirrhosis to liver biopsy specimens: Interobserver agreement. Pathology International, 60(3), 167-174] [25]* presented in Table 2. A) Fibrosis stage 0: No portal fibrosis or fibrosis limited to portal tracts. B) Portal fibrosis with periportal fibrosis or incomplete septal fibrosis. C) Bridging fibrosis with variable lobular disarray. D) Liver cirrhosis with regenerative nodules and extensive fibrosis.


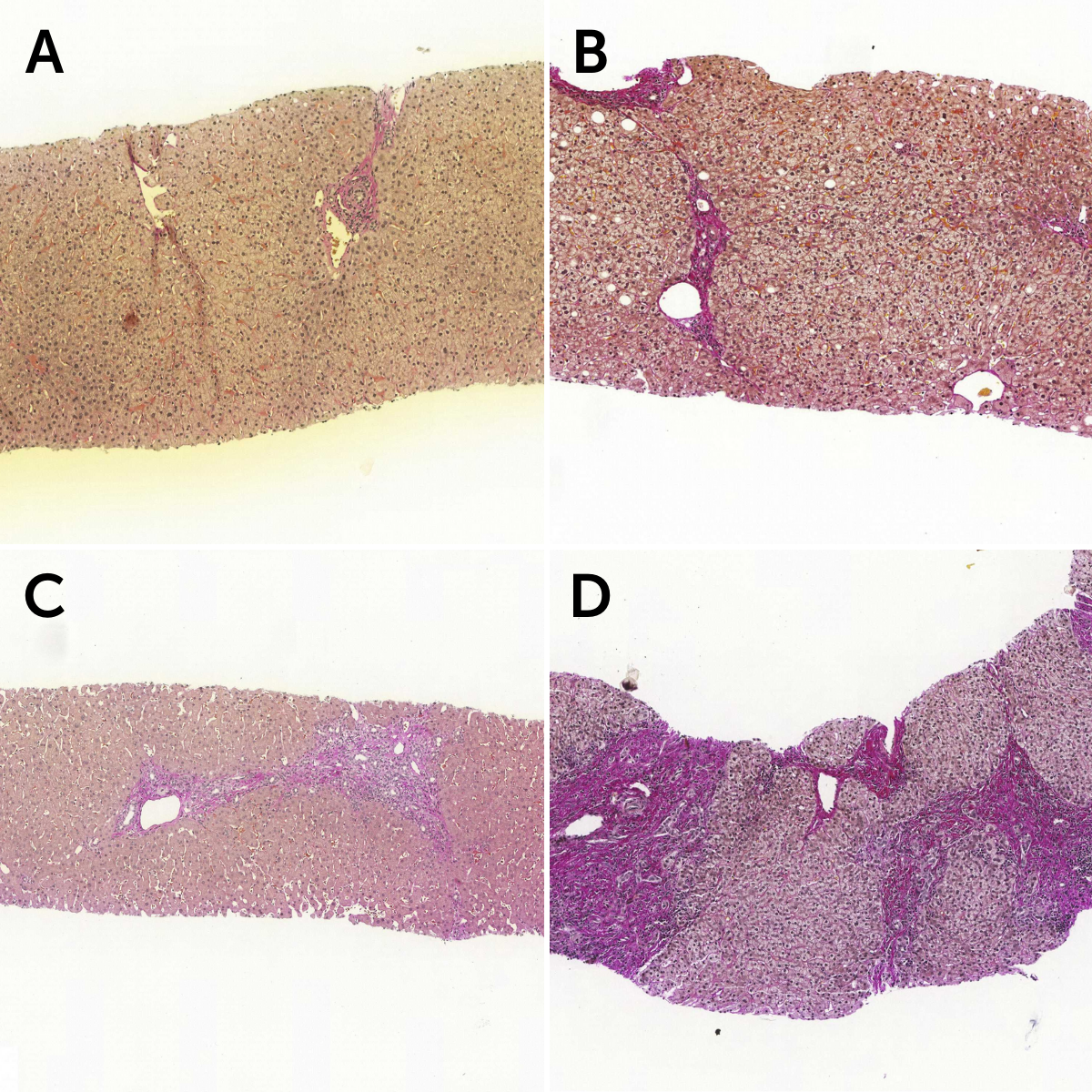

Supplement: Supplementary file 2 — Additional file 2. [file 13000_2021_1102_MOESM2_ESM.zip › Appendix B.docx]
