# Supplementary figures and images for: Chronic cholestasis detection by a novel tool: automated analysis of cytokeratin 7-stained liver specimens
Source: Diagn Pathol. 2021 May 6;16:41. doi: 10.1186/s13000-021-01102-6 (PMC8101247; doi:10.1186/s13000-021-01102-6)

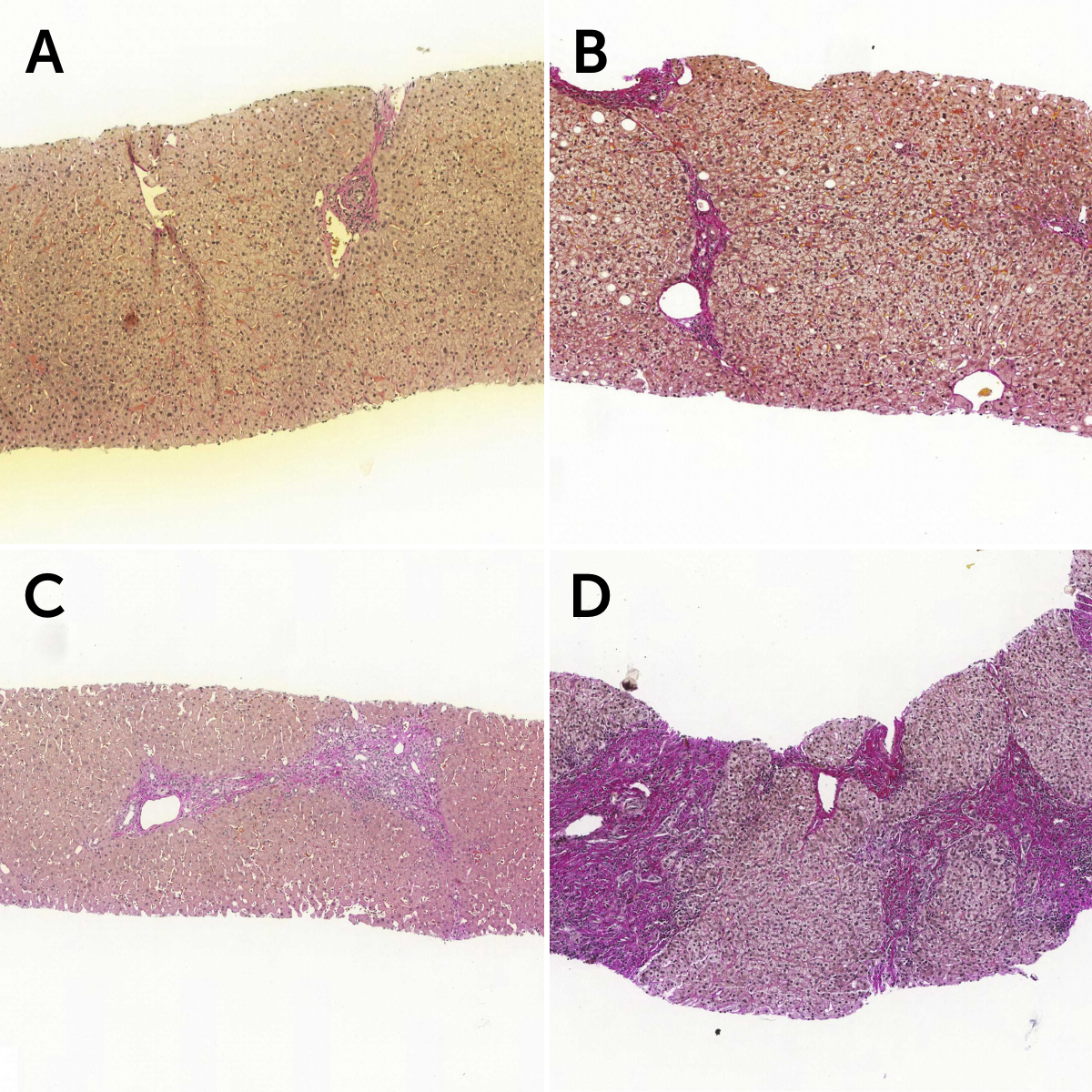

Supplement: Supplementary file 2 — Additional file 2. [file 13000_2021_1102_MOESM2_ESM.zip › Appendix B, Figure 1_pdf.png]

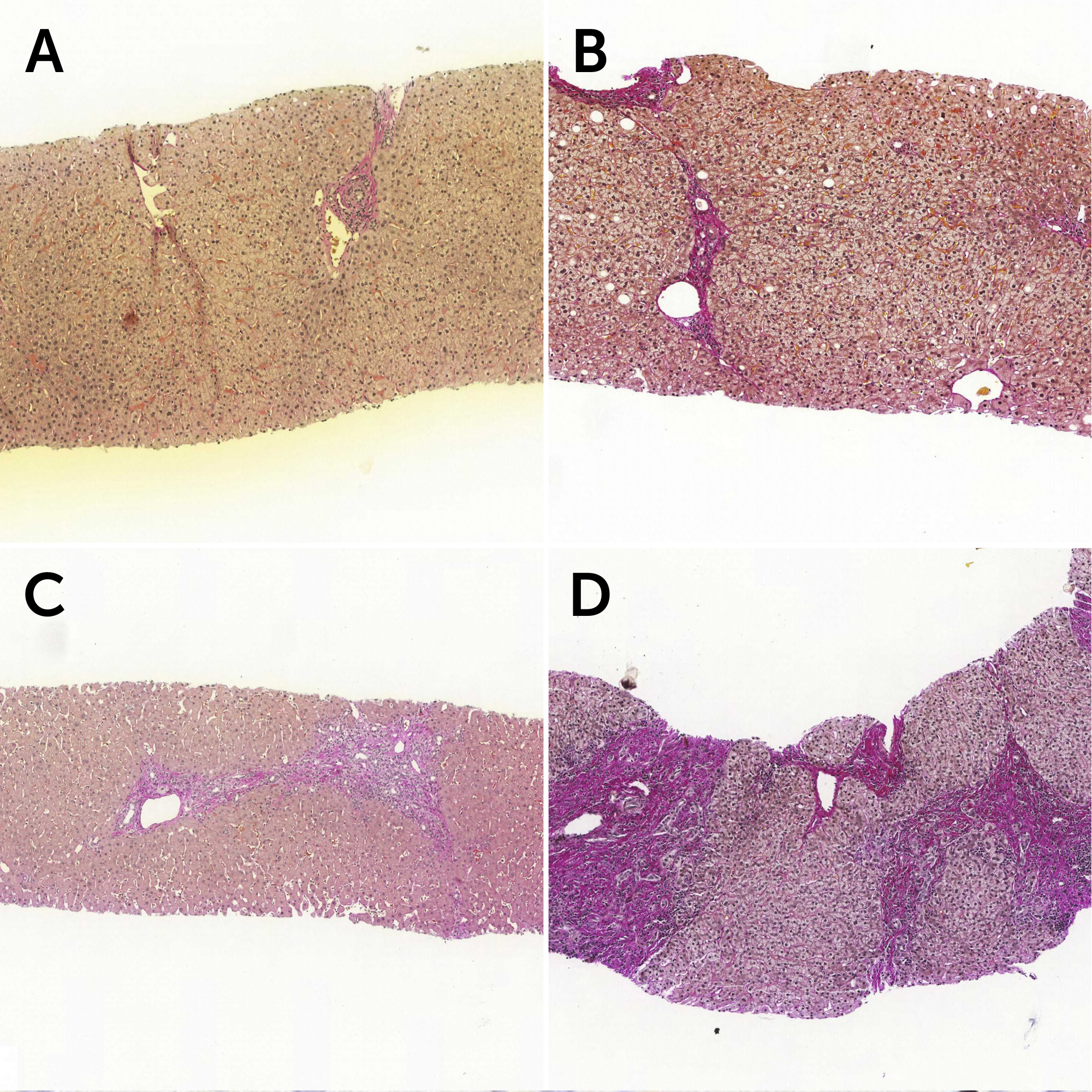

Supplement: Supplementary file 2 — Additional file 2. [file 13000_2021_1102_MOESM2_ESM.zip › Appendix B, Figure 1_web.png]
